# Molecular mimicry, inflammatory bowel disease, and the vaccine safety debate

**DOI:** 10.1186/s12916-014-0166-6

**Published:** 2014-09-18

**Authors:** Susy Yusung, Jonathan Braun

**Affiliations:** Department of Pediatrics, Harbor-UCLA Medical Center, 1000 W Carson St, Torrance, CA 90502 USA; Department of Pathology and Laboratory Medicine, UCLA, 10833 Le Conte Ave, Los Angeles, CA 90095 USA

**Keywords:** Crohn’s Disease, Ulcerative Colitis, humoral immunity, epitope spreading

## Abstract

Preventive immunization has provided one of the major advances in population health during the past century. However, a surprising cultural phenomenon is the emergence of concerns about immunization safety, in part due to prominently controversial biomedical studies. One ongoing theoretical safety concern is the possibility of human molecular mimicry by measles, mumps, rubella (MMR) antigens. The study of Polymeros *et al*. in this *BMC Medicine* presents a systematic evaluation and refutation of this safety concern. This provides significant new scientific evidence in support of the safety of pediatric vaccines, which will inform the ongoing policy and cultural understanding of this important public health measure.

Please see related research article: http://www.biomedcentral.com/1741-7015/12/139/abstract.

## Background

Measles virus is a highly contagious organism that can cause multiple organ system complications and even death. Introduction of the measles, mumps, rubella (MMR) vaccine in the 1970s has saved innumerable lives and attenuated severe morbidities globally. Despite these remarkable gains, a claim emerged in the 1990s which associated MMR vaccine with colitis and autism spectrum disorders [1-4]. These association studies have been recognized as methodologically flawed and in some instances factually erroneous by the scientific and medical communities [5]. However, doubt and even outright rejection of the vaccine’s safety still linger in various niches of our society. These concerns have also reverberated in the inflammatory bowel disease (IBD) community, in part related to the publication of a study series implying measles infection or vaccination with the live attenuated virus mediate pathogenesis of Crohn’s disease (CD) [3,4,6]. Despite recognition that these studies were flawed in methodology and hypothesis, they continue to provoke much debate and inquiry into the role of measles virus in the pathogenesis of IBD.

### Biologic context

Exposure to environmental antigens has long been suspected to induce development of IBD in genetically susceptible individuals. The ease of transmission and pervasiveness of certain infectious organisms makes the possibility of micro-organisms as antigenic triggers of IBD into a tantalizing hypothesis. The discovery that certain bacterial communities instigate inflammation of the gut mucosa further intensified research of the microbiome and simultaneously heightened awareness of elegant mechanisms by which viruses may serve as potential regulators of gut mucosal inflammation [7]. In this context, a number of viruses have been studied in association with human CD - measles virus being one of the most commonly studied, preceded by cytomegalovirus and Epstein-Barr virus [8].

Several groups have investigated the plausible link between measles and IBD either through epidemiological studies or biological analysis of tissue specimens. A study published in 1995 first suggested that recipients of measles vaccination had a three-fold increased risk of developing CD and ulcerative colitis (UC) compared to unvaccinated controls; however, this study was found to be limited by methodological flaws and could not be validated [6,9]. Carefully designed studies thereafter demonstrated no increased risk of CD in children vaccinated with MMR [10,11]. Studies also comparing the frequency of measles seropositivity in CD, UC and healthy populations found no significant differences between all three groups [12,13]. In addition to these epidemiological studies, efforts have been made to detect the presence of persistent measles virus in tissue or serum samples from IBD patients using PCR and immunohistochemical techniques. Results of those immunohistochemical studies have been discordant and contradictory [2,14], while the highly sensitive reverse PCR method failed to detect any measles virus genome in the intestinal tissues of CD patients [15-17]. In light of these studies, several investigators have proposed a ‘measles related antigen’ in the human intestine which shares identical epitopes with the measles virus and induces immune reactivity through mechanisms of molecular mimicry [18-20].

In a study published in *BMC Medicine*, Polymeros *et al*. [21] revisit the issue of measles-induced molecular mimicry as a mechanism which directs immune dysregulation in CD. Firstly, this study is distinguishable from prior studies by virtue of combined employment of experimental immunologic data, bioinformatics, and detailed molecular analysis of measles virus and human antigens. Results from prior immunological studies have demonstrated the development of antibodies against HEMA and VGLF antigens following vaccination, isolating these viral proteins as candidates for intestinal antigen mimicry. The antigenic potential of HEMA and VGLF epitopes are verified using prediction algorithms and, subsequently, human intestinal proteins which share these antigenic epitopes are identified and analyzed as potential viral mimic candidates. Thus, the utilization of technically validated methods and algorithms serve as platforms for identification of candidate mimic proteins.

The paper identifies several candidate intestinal peptides as potential measles virus antigen mimics. Changes in the expression of some of the peptides, such as intestinal fatty acid binding protein and sucrase isomaltase, have been previously associated with intestinal inflammation in CD patients. Another protein TOG, had been previously identified as sharing sequence analogue with *Mycobacterium avium paratuberculosis* (MAP), and antibodies reactive to this protein were found in both CD and healthy controls [22]. Although these intestinal proteins appear to be viable measles mimic candidates, no significant differences in reactivity to any of these antigens were found among CD, UC and healthy controls. Moreover, there were no significant differences in reactivity to highly antigenic epitopes of HEMA or VGLF among CD, UC and healthy individuals. These results are in contrast to previous studies by the present investigators, which found cross-reactivity between two sets of MAP and human intestinal peptides although the observed cross reactivity was well within the random rates predicted for heterologous peptides. In comparison, the lack of any cross reactivity between measles and human intestinal peptides reinforces the high unlikelihood of the measles virus having a role in CD pathogenicity via the mechanism of molecular mimicry.

## Conclusions

At a biologic level, the present study of Polymeros *et al*. thus is a fresh test of the hypothesis of microbial molecular mimicry in the pathogenesis of IBD, and provides thoughtful and fastidious evidence against this hypothesis. As we have noted, molecular mimicry is the putative link of concern between MMR vaccine and IBD risk. Accordingly, at the level of public safety and policy, this paper provides important new evidence against this link. This carefully performed study should be an important new reassurance to concerned families and medical caregivers on the safety of the MMR vaccine with respect to IBD susceptibility.
